# A Secure RFID Authentication Protocol Adopting Error Correction Code

**DOI:** 10.1155/2014/704623

**Published:** 2014-05-18

**Authors:** Chien-Ming Chen, Shuai-Min Chen, Xinying Zheng, Pei-Yu Chen, Hung-Min Sun

**Affiliations:** ^1^School of Computer Science and Technology, Harbin Institute of Technology Shenzhen Graduate School, Shenzhen 518055, China; ^2^Shenzhen Key Laboratory of Internet Information Collaboration, Shenzhen 518055, China; ^3^Department of Computer Science, National Tsing Hua University, Hsinchu 300, Taiwan

## Abstract

RFID technology has become popular in many applications; however, most of the RFID products lack security related functionality due to the hardware limitation of the low-cost RFID tags. In this paper, we propose a lightweight mutual authentication protocol adopting error correction code for RFID. Besides, we also propose an advanced version of our protocol to provide key updating. Based on the secrecy of shared keys, the reader and the tag can establish a mutual authenticity relationship. Further analysis of the protocol showed that it also satisfies integrity, forward secrecy, anonymity, and untraceability. Compared with other lightweight protocols, the proposed protocol provides stronger resistance to tracing attacks, compromising attacks and replay attacks. We also compare our protocol with previous works in terms of performance.

## 1. Introduction

RFID (radio frequency identification) is a technique used for identifying objects via radio frequency. It has become very popular in many applications such as access control systems, supply chain management systems, transportation, ticketing systems, and animal identification. The global transaction of RFID system was US$2.65 billion in 2005 [1] and increased to US$5.56 billion in 2009 [2]. At present, RFID technology has become one of the fastest growing markets in radio communication industries.

The RFID systems are composed of three components: a set of tags, RFID readers, and one or more backend servers. A backend server is responsible for storing the related information of tags, calculating the computational processes when authenticates a tag; in addition, a backend server is usually with a more powerful computation ability than RFID reader and tags. An RFID reader (called a reader in this paper) can access the backend server via secure network channel and then acquire the information related to the tags. Generally, backend servers and readers are treated as a whole entity since they are usually connected with each other through a wired line. RFID tags are small electronic devices composed of antennas, microprocessors, and memory storages. A tag can communicate with a reader by using radio frequency signals transmitting from the reader. Normally, RFID tags can be classified into three types: active tag, semiactive tag, and passive tag. Active tags contain batteries that can actively communicate with the readers. Semiactive tags also have batteries, but they will remain silent until they receive query from a reader. Passive tags contain no battery, and their energies come from the reader's radio signals through antennas. Regarding the cost of the tag, the active and semiactive tags are expensive and each costs about US$20, while the passive tags are usually considered as* low-cost RFID tags* which cost about US$0.05 each. Since RFID tags usually play the roles as tickets or ID cards, most of the RFID-tagged products are small and portable, and people carry them in their daily life. For example, the e-passports combine traditional paper passports and embedded RFID chips which contain personal biometric information. They are carried by travelers from over 60 countries in the world.

While RFID technology offers convenience, security and privacy issues are still the number one concern of most RFID applications today. Since an RFID tag can be continuously scanned within a 10 meter radius, the tag carrier's location can be easily traced without awareness; thus privacy becomes an important issue in RFID applications. Moreover, RFID tags may contain sensitive information about the carrier in which the information should not be revealed to anyone, especially to an attacker. In other words, tags should first authenticate the reader's validation before sending private data. Meanwhile, readers should also be able to authenticate tags to prevent counterfeit tags.

To address these problems, researchers have proposed many RFID protocols to achieve mutual authentication, untraceability, and other security requirements. However, with limited computational ability and insufficient memory storage on its embedded chip, low-cost RFID protocol design still remains a challenge. Previous studies showed that the number of logic gates available for security functionality on a low-cost RFID tag is 400 to 4000 [3], which is not enough to implement most public key or symmetric key cryptosystems. Therefore, an RFID protocol should be as computationally lightweight as possible.

In this paper, we propose a lightweight mutual authentication protocol based on error correction codes to provide a secure RFID mechanism. More specifically, our protocol provides mutual authenticity and untraceability to protect the security and privacy of tag carriers. We also present an evaluation on the security and performance level of our proposed protocol. Compared to other previous works, our protocol not only meets the fundamental security requirements but is also lightweight enough to be implemented on low-cost RFID tags.

The rest of this paper is organized as follows.  Section 2 reviews the related works of RFID protocols.  Section 3 describes a brief introduction of the error correction codes used in this paper. Our proposed RFID mutual authentication protocol is presented in Section 4. In Section 5, we analyze the security constraints of our protocol, followed by an evaluation of the performance of our protocol in Section 6. Finally, a conclusion is given.

## 2. Related Work

With the rapid growth of network technology, security issues have been a matter of concern in various network environments [4–12] such as wireless sensor networks, social networks, and Internet of Things. In the RFID environment, security and privacy issues also receive increasing attention recently.

There are many RFID protocols using one-way hash functions (e.g., [13, 14]) to perform their authentication process by hashing random challenges, tag identity, and/or secret key into one message. However, hardware implementations of hash functions such as SHA-1 and MD5 are generally considered too expensive to be implemented on low-cost RFID tags. However, literatures [3, 15] describe some of these implementation issues in which some of them proposed their lightweight hash functions that can be implemented on low-cost RFID tags. These lightweight hash functions include Tav-128 proposed by Peris-Lopez et al. [16], low-cost SHA-1 proposed by O'Neill [17], and H-PRESENT-128 proposed by Bogdanov et al. [18].

The RFID authentication protocol can be classified into 4 classes. The first class refers to those protocols that apply conventional cryptographic functions, such as symmetric encryption or public key algorithm. The second class refers to those protocols that apply random number generator and one-way hash function. The third class refers to those protocols that apply random number generator and cyclic redundancy code (CRC) checksum. The last one refers to those protocols that apply simple bitwise operations (such as XOR, AND, OR, etc.). Generally, the third class is treated as lightweight level. Although our protocol has to adopt one hash function, we can simply apply the lightweight hash functions mentioned in the previous paragraph to achieve the goal of lightweight computation. Hence, by applying those lightweight hash functions, we propose our lightweight RFID protocol.

Lightweight authentication protocols aim to achieve mutual authentication through simple operations like bitwise XOR and binary addition. In 2005, Juels and Weis proposed a multiround lightweight authentication protocol called HB^+^ [19], which is an improvement of* HumanAut*, a human-to-computer authentication protocol designed by Hopper and Blum [20]. Nevertheless, Gilbert et al. proved that the HB^+^ protocol is vulnerable to a man-in-the-middle attack [21]. There are currently many improvements of the HB^+^ protocol, for example, the HB^++^ protocol proposed by Bringer et al. in 2006 [22], the HB-MP protocol proposed by Munilla and Peinado in 2007 [23], and the HB# protocol proposed by Gilbert et al. in 2008 [24].

The EPCglobal Class 1 Generation 2 UHF Air Interface Protocol Standard (generally known as Gen2 standard) [25] is a standard that defines the physical and logical requirements of RFID systems. In Gen2 standard, an RFID tag maintains the computational abilities to perform simple bitwise operations, 16-bit cyclic redundancy checks (CRC) and 16-bit pseudorandom number generator (PRNG) function. In 2009, Sun and Ting presented the *Gen*2^+^ protocol [26] for Gen2 standard. In this protocol, each tag stores a string called key pool, which is shared with a backend server. *Gen*2^+^ protocol is appropriate for Gen2 standard; however, Burmester et al. demonstrated an attack to break this protocol in 2009 [27].

## 3. Preliminary 

In information theory and coding theory of computer science, error correction code (ECC) is a technique that enables the communication parties to correct the transmission errors which are incurred by the channel noise. This technique has been studied over 50 years, and substantial coding algorithms are proposed. In the following, we provide a brief introduction to one of the subclasses of ECC, called a linear block codes; in addition, if a linear block code fulfills some properties, it will form a special case of linear block codes, called perfect code. We will have a short description of perfect code in the end of this section as well.

### 3.1. Linear Block Codes

During the transmission, the information source, or sender, will encode a *k*-bit message blocks into *n*-bit* codewords* by using channel encoding algorithm, where *n* > *k*. There are total 2^*k*^ distinct messages and corresponding 2^*k*^ distinct codewords. These 2^*k*^ fixed length codewords are called a set of block codes and is denoted by *C*(*n*, *k*). A *C*(*n*, *k*) block code is called linear block code if it satisfies Definition 1.

**Definition 1**

A block code of 2^*k*^ codewords of each *n*-bit in length is called a linear block code if and only if these 2^*k*^ codewords form a *k*-dimension vector subspace over the Galois Field GF(2).

Because a linear block code *C*(*n*, *k*) is a *k*-dimension vector subspace, it is possible to find *k* linearly independent codewords in *C*(*n*, *k*) that every codeword in *C*(*n*, *k*) is a linear combination of these *k* codewords. We write these codewords into *k* row vectors *g*_0_, *g*_1_,…, *g*_*k*−1_ and form a *k* × *n* matrix *G* as follows:

(1) $$\begin{array}{c} G=\left[\begin{array}{c} g_{0}\\ g_{1}\\ \vdots \\ g_{k-1} \end{array}\right]=\left[\begin{array}{cccc} g_{0,0} & g_{0,1} & \ldots & g_{0,n-1}\\ g_{1,0} & g_{1,1} & \ldots & g_{1,n-1}\\ \vdots & \vdots & & \vdots \\ g_{k-1,0} & g_{k-1,1} & \ldots & g_{k-1,n-1} \end{array}\right], \end{array}$$

where *g*_*i*_ = (*g*_*i*,0_, *g*_*i*,1_,…, *g*_*i*,*n*−1_) for 0 ≤ *i* ≤ *k* − 1. For a message *m* = (*m*_0_, *m*_1_,…, *m*_*k*−1_), the corresponding codeword *v* can be computed as follows:

(2) $$\begin{array}{c} v=m\cdot G=\left(m_{0},m_{1},\ldots ,m_{k-1}\right)\cdot \left[\begin{array}{c} g_{0}\\ g_{1}\\ \vdots \\ g_{k-1} \end{array}\right]. \end{array}$$

To decode a codeword, we first construct a (*n* − *k*) × *n* matrix *H*, which is composed of *n* − *k* linearly independent rows such that any linear combination of rows in *G* is orthogonal to the rows of *H*. This implies that any codeword *v* in *C*(*n*, *k*) generated by *G* must satisfy the following property.

**Definition 2**

A vector *v* is a codeword in *C*(*n*, *k*) generated by *G* if and only if *v* · *H* ^*T*^ = 0.

Let *r* = *v* + *e* be the received message, where *v* is the codeword and *e* = (*e*_0_, *e*_1_,…, *e*_*n*−1_) is the error vector incurred by the channel noise. For a received message *r*, the receiver first computes a (*n* − *k*)-bit vector *s* = *r* · *H* ^*T*^ = (*s*_0_, *s*_1_,…, *s*_*n*−*k*−1_) called syndrome, which can be calculated as *s* = *r* · *H* ^*T*^ = (*v* + *e*) · *H* ^*T*^ = *v* · *H* ^*T*^ + *e* · *H* ^*T*^ = *e* · *H* ^*T*^. If there is no error, the syndrome *s* will be zero and the receiver recognizes that *r* is the correct codeword. Nonetheless, if *s* is nonzero, the receiver has to determine the error vector *e* from *s*. The methods to find the error vector are different according to each coding algorithm, but we can always put every possible error pattern into the computation, get the corresponding syndromes, and construct a lookup table for the receiver in advance. Once the receiver obtained an error vector, it can recover the original codeword by computing *v* = *r* + *e*.

Hamming weight of a binary vector is defined as the number of 1 in the vector. We further define Hamming weight function Hw(·) to be a function that returns the Hamming weight from an input vector. Hamming distance is the number of positions that two vectors differ from each other, denoted as Dis(·). For instance, let *v* = 1011 and *u* = 0110 be two binary vectors; then Dis(*v*, *u*) = 3 since they differ in the first, second, and fourth positions.

The error correcting ability of a linear block code depends on the minimum Hamming distance (denoted as *d*) of every two codewords. We denote *C*(*n*, *k*, *d*) as an error correction code where its codeword length, message length, and minimum Hamming distance are *n*, *k*, and *d*, respectively. A *C*(*n*, *k*, *d*) code is capable of correcting all the error vectors which have the Hamming weight less than or equal to *t* = ⌊(*d* − 1)/2⌋.

### 3.2. Perfect Code

For a *C*(*n*, *k*, *d*) code, there are 2^*k*^ codewords each with a *n*-bit length, and each codeword might have errors that occurred in *t* positions at most. Therefore, there will have total $2^{k}\times \sum_{i=0}^{t}\left(\begin{array}{c} n\\ i \end{array}\right)$ messages that can be corrected to be a valid codeword in *C*(*n*, *k*, *d*). Typically, this number is no greater than the number of totally 2^*n*^ possible messages. If *C*(*n*, *k*, *d*) satisfies $2^{k}\times \sum_{i=0}^{t}\left(\begin{array}{c} n\\ i \end{array}\right)=2^{n}$, it is called a* perfect code*. That is, every possible message can be corrected to be a valid codeword.

## 4. The Proposed Protocols

In this section, we propose a lightweight RFID authentication protocol. Our main idea is to provide a mutual authentication between reader and tag. Our protocol is designed for low-cost RFID tags; therefore, the requirement for implementing our protocol will not overload the capabilities of the tags. Besides, we also propose an advanced version of our protocol to provide key updating.

Our protocol is suitable for large scale RFID systems, such as ticketing systems, transportation systems, and supply chain systems. These applications are generally composed of millions of RFID tags and readers. More importantly, the proposed protocol is appropriated for the reader to find out a specific tag from a large group of tags. For example, an airport employee desires to find a specific RFID tagged luggage from a loaded cargo truck. The proposed scheme checks whether the specific tag is in this area. In these large scale systems, readers are normally held by authorized persons or are used under supervision. They can easily connect to servers and synchronize their data. The tags in these systems are generally carried by humans or attached to goods and baggage. They are frequently scanned by the valid readers, and, in some situations, the tags can be brought back to a secure check (e.g., the RFID tagged tickets can be recycled). Before introducing the proposed protocol, the notations used are presented in the Notation section at the end of the paper.

### 4.1. Initialization

Initially, the administrator generates a pseudorandom number generator *g*(·), a one-way hash function *h*(·), and a *C*(*n*, *k*, *d*) error correction code, with the *k* × *n* generator matrix *G* and the (*n* − *k*) × *n* parity check matrix *H*. Each tag, denoted as *T*_*i*_, *i* = 0,1,…, has its unique identifier. We also denote their identifiers as *T*_*i*_ just for simplicity. For each tag *T*_*i*_, the backend server *S* randomly generates secret keys *k*_*i*_. Let *s*_*i*_ be a *k*-bit long binary vector which is a possible syndrome pattern induced by *H*. Each tag *T*_*i*_ is assigned with a syndrome pattern *s*_*i*_. Then, *S* stores the tags' identifiers, *T*_*i*_ and corresponding *k*_*i*_ and *s*_*i*_ in its database. Finally, *S* writes *g*(·), *h*(·), *T*_*i*_, *k*_*i*_, *s*_*i*_, *G*, and *H* into the storage memory of tag *T*_*i*_ in a secure environment (e.g., at RFID tags manufacturer). For every authorized reader, *S* also writes *g*(·), *h*(·), *T*_*i*_, *k*_*i*_, *s*_*i*_, *G*, and *H* into their storage memory.

### 4.2. Authentication Protocol: Basic Version

The main objective of this protocol (Algorithm 1) is to establish a mutual authentication relationship between a reader *R* and a specific target tag *T*_*x*_ in a group of tags. Since the reader may receive a substantial amount of tags' responses for a single query, our protocol adds a filtering mechanism based on error correction codes to prevent the reader from having to examine every responding message.

At the beginning, *R* selects its target tag, *T*_*x*_, and retrieves the corresponding *k*_*x*_ and *s*_*x*_ from database. In step 1, *R* randomly generates a codeword in *C*(*n*, *k*, *d*), denoted as *C*_*R*_. Then *R* generates an error vector *e*_*R*_ with Hamming weight less than or equal to *t* = ⌊(*d* − 1)/2⌋, which is the maximum error correcting ability of *C*(*n*, *k*, *d*). Finally, *R* computes the masked codeword *C*_*R*_′ by adding *C*_*R*_ with *e*_*R*_. The error vector generated in this step must be selected carefully so that the syndrome derived from *C*_*R*_′ will equal the preassigned pattern *s*_*x*_.

In step 2, *R* broadcasts a query to tags, along with a random challenge *N*_*R*_ and the masked codeword *C*_*R*_′. In Step 3, the tags attempt to decode *C*_*R*_′ with the parity check matrix *H* and compute a syndrome *s* = *C*_*R*_′ · *H* ^*T*^. If a tag *T*_*i*_ finds that *s* is equal to the pattern stored in its storage memory, it randomly generates a codeword *C*_*i*_ in *C*(*n*, *k*, *d*) and a challenge *N*_*i*_. Then *T*_*i*_ computes a verifier message *V*_*i*_ = *g*(*s* ⊕ *N*_*R*_ ⊕ *h*(*N*_*i*_ ⊕ *k*_*i*_)) and the masked codeword *C*_*i*_′ = *C*_*i*_ + *e*_*i*_, where *e*_*i*_ is a random error vector with Hw(*e*_*i*_) ≤ *t*. Since *s* is shorter than *N*_*R*_, *s* should be padded before XORing with *N*_*R*_. For the other tags that cannot find *s* in its preassigned pattern, the verifier message *V*_*i*_ and masked codeword *C*_*i*_′ are set to a random value.

Finally, no matter what their preassigned syndromes are, the tags respond *k*_*i*_ ⊕ *C*_*i*_′, *V*_*i*_, and *N*_*i*_ to the reader in step 4. Note that the masked codeword *C*_*i*_′ is further masked with the key *k*_*i*_ to prevent possible tracing attack.

In Step 5, *R* authenticates *T*_*i*_ by examining the received messages. First, *R* uses *k*_*x*_ to unmask (XOR with *k*_*x*_) the received messages and tries to decode every masked codeword *C*_*i*_′. If *R* finds a codeword that cannot be decoded with the decoding algorithm, *R* simply ignores it and proceeds to the next masked codeword. Since the nontarget tags will always generate uncorrectable masked codewords, this method will filter out all the unnecessary messages sent by the nontarget tags, which reduces the computational loads of *R*. If one of these masked codewords *C*_*i*_′ sent by *T*_*i*_ can be decoded, *R* uses the stored secret keys *k*_*x*_, *N*_*R*_, *N*_*T*_, and *s* to verify if the corresponding *V*_*i*_ is sent from *T*_*i*_. If *V*_*i*_ is correct, *R* computes another verifier message *V*_*R*_ = *g*(*s* ⊕ *N*_*i*_ ⊕ *h*(*N*_*R*_ ⊕ *k*_*i*_)). Since *s* is shorter than *N*_*i*_, *s* should be padded before XORing with *N*_*i*_. At this step, *R* has authenticated *T*_*i*_ to be the target tag *T*_*x*_. If either *C*_*i*_′ cannot be decoded or *V*_*i*_ is incorrect, *R* will not recognize *T*_*i*_ as its target, so *R* assigns *V*_*R*_ a random value. Whether *T*_*i*_ is the target tag or not, *R* always sends *V*_*R*_ to *T*_*i*_ (step 6).

In step 7, *T*_*i*_ verifies the received *V*_*R*_ to authenticate *R*. Only the target tag *T*_*x*_ that has the key *k*_*x*_ can accept *V*_*R*_ as the valid message and authenticate *R* by using *k*_*x*_, *N*_*i*_, *N*_*R*_, and *s*. At this step, both *R* and *T*_*i*_ have authenticated each other.

### 4.3. Error Vector Selecting

As we stated before, the error vector generated by the reader must be selected carefully so that *T*_*x*_ can derive a syndrome that equals the preassigned syndrome pattern *s*_*x*_. It is straightforward since the syndromes are originally used by decoding algorithms to find corresponding error vectors. That is, *R* can simply use the decoding algorithm to find the corresponding error vector of a specific syndrome. This error vector is exactly the error vector that should be used to mask the codeword generated by *R* in the first step.

### 4.4. Session Key

Typically, the reader and the tag would exchange data after completing the authentication process. These data are sometimes considered private; for example, the tag used in a hospital would contain the records of its carrier. The threat of eavesdropping attacks makes the tag carriers feel insecure about transmitting sensitive data. To address this problem, we construct a mechanism to establish a session key and use it to encrypt the sensitive data. We suggest that the reader and the tag use the session key *sk* = *g*(*k*_*i*_ ⊕ *N*_*R*_ ⊕ *N*_*i*_) to encrypt the messages. Without the secret key *k*_*i*_, the adversary cannot decrypt the session *sk* to break the encrypted messages.

### 4.5. Secret Key Update

The secret key should not be used permanently. In fact, if the key is compromised, the messages encrypted with this key are also compromised. Hence, both the probability of messages compromised and the probability of financial loss will increase with the length of time in which a key is in use. We think that the secret keys stored in the readers and the tags should update regularly. Previous works use two approaches to perform this updating procedure. One possible approach is to have tags carriers bring their tags back to an authorized institution so that the new keys can be written into the tags in a secure environment. Another approach is to have the tags use the one-way hash functions stored in them to calculate new keys by hashing the older one.

The first approach could be combined with our authentication protocol in some RFID systems like ticketing systems and supply chain systems, since the tags are generally returned to the backend server. The second approach is also adequate for our protocol. Both the tag and the reader can hash their current secret key *k*_*i*_ into the new one after a successful authentication process. More precisely, the tag will update its secret key after verifying *V*_*R*_ at step 7, and the reader will update its key before sending *V*_*R*_ to the tag (step 6). We suggest the entities update the key by computing *h*(*k*_*i*_||*N*_*R*_||*s*), where || denotes the string concatenation operation. The new secret key *k*_*i*_ is then assigned to this hashing value. Note that the session key construction process should be performed prior to updating the secret key.

If the tag does not receive the verifier message *V*_*R*_, the keys between the reader and the tag might be desynchronized. This means that next time this tag's verifier message will be rejected by the reader. To address this problem, the reader should store the previous key before updating. Once the reader discovers that *C*_*i*_′ can be decoded but *V*_*i*_ is incorrect, it can attempt to verify the message by using the older key. This mechanism can help the system resist desynchronization attacks.

### 4.6. Advanced Protocol: With Secret Key Update

Now we present a modification of our protocol with the secret key updating mechanism in it. The steps of the modified protocol are depicted in Algorithm 2. The terms *k*_*i*_^cur^ and *k*_*i*_^old^ represent the current secret key and the previous secret key for *T*_*i*_. Note that the value *k*_*i*_ stored in the tag may be either *k*_*i*_^cur^ or *k*_*i*_^old^. After a successful authentication process, the reader constructs the session key by using either *k*_*i*_^cur^ or *k*_*i*_^old^, depending on which key is used to authenticate the tag. And the tag constructs the session key by using *k*_*i*_. Then, the reader updates its secret keys by setting *k*_*i*_^cur^ = *k*_*i*_^new^ and *k*_*i*_^old^ = *k*_*i*_^cur^, while the tag updates the secret key by setting *k*_*i*_ = *h*(*k*_*i*_||*N*_*R*_||*s*).

Our protocol provides a convenient method for the tag and the reader to authenticate each other before exchanging data. Since the reader will receive many messages sent from other tags at the same time, our protocol uses the properties of error correction code to filter out the unnecessary messages. Therefore, the computational load of the reader is reduced. After mutual authentication, the relation between the reader and the tag is established. They will both update their secret keys to the new ones in order to defend against possible attacks. Furthermore, the two entities can also construct a session key to protect the message transmitted later.

## 5. Security Analysis

In this section, we show that our protocols fulfill the security requirements for RFID systems.

### 5.1. Mutual Authenticity

A reader can easily authenticate the tag's identity since only the valid tag has the secret key needed to construct the correct verifier message. The random challenge *N*_*R*_ sent by the reader prevents the attackers from pretending to be the target tag and thus it ensures reader-to-tag authenticity. Since the reader must authenticate itself to the server before retrieving any keying information from the server, the tag can trust the reader who has the correct secret key. In other words, tag-to-reader authenticity is achieved indirectly via server-to-reader authenticity.

### 5.2. Integrity

The integrity of the exchanged messages is guaranteed since the messages are encrypted by the session keys. The modification of these messages will produce meaningless plaintext, and both reader and tag can detect such modifications. During the authentication process, the adversary can also eavesdrop and modify the exchanged messages. Nevertheless, any modification on *k*_*i*_ ⊕ *C*_*i*_′, *V*_*i*_, or *V*_*R*_ will lead to an incorrect verifying result on either the reader or the tag. When an adversary attempts to modify the random challenge *N*_*i*_, the reader can still find the inconsistencies of *N*_*i*_ and *V*_*i*_ and thus reject the message. However, the modification of *C*_*R*_′ and *N*_*R*_ cannot be discovered by the tags because these messages are independent. This modification causes the tags to produce incorrect responses. But since the modification on *C*_*R*_′ will change its underlying *s*, all the verifier messages *V*_*i*_ are invalid to the reader. These messages cannot be used to perform any further attacks on the RFID system. Although we cannot guarantee the integrity of *C*_*R*_′ and *N*_*R*_, the result of the modification on these messages is nothing but a denial-of-service attack.

### 5.3. Forward Secrecy

Our protocols maintain forward secrecy. Since the keys were updated by using one-way hash function in every session, the attacker cannot acquire the previous secret keys used in the prior sessions. Therefore, the previous session keys and the exchanged messages are secure.

### 5.4. Anonymity and Untraceability

Our protocols do not leak the tag's identifier or any sensitive information. Therefore, our protocols fulfill the requirement of anonymity. During the authentication protocol, *T*_*i*_ will send messages *k*_*i*_ ⊕ *C*_*i*_′, *V*_*i*_, and *N*_*i*_ to *R*. The adversary is able to eavesdrop all the messages sent from its target tag. With the help of these collected messages, if the adversary is able to distinguish the target tag's messages from the other tags' messages, it is able to trace this tag. Obviously, the random challenge *N*_*i*_ is indistinguishable from any other random number, so the adversary cannot use it to trace the tag. The verifier message *V*_*i*_ is constructed by a PRNG with *N*_*i*_ as its seed; thus it is also a random number.

Every tag stores the same generator matrix; therefore, all of them share the same probability of producing the same codeword. However, different tags will add different error vectors. As a result, the masked codewords produced by some tags can be decoded correctly while the others cannot. Once the parity check matrix is known by the adversary, this property may be used by the adversary to trace the tag. To defend against this, the tags further mask their messages with the secret keys. The adversary cannot apply decoding algorithm to the messages without first unmasking them. Hence, we can guard against tracing attacks as long as the target tag's key is secure.

### 5.5. Confidentiality

Now we analyze the probability that an attacker will successfully guess one secret key of a tag with different advantages provided. First, if the adversary knows no additional information, the success probability is surely 1/2^*n*^. If the adversary acquires generator matrix *G* by compromising a tag or a reader, it will have some advantages in constructing the codewords. Now the adversary attempts to guess the *C*_*i*_′ to derive *k*_*i*_ from the message *k*_*i*_ ⊕ *C*_*i*_′ sent in step 4 of the proposed protocol. The number of all valid codewords *C*_*i*_ is 2^*k*^. With the error vector *e* added in which Hw(*e*) ≤ *t*, the number of all possible *C*_*i*_′ = *C*_*i*_ ⊕ *e* is $\sum_{i=0}^{t}\left(\begin{array}{c} n\\ i \end{array}\right)\times 2^{k}$. Therefore, the success probability of guessing the correct *C*_*i*_′ and *k*_*i*_ is $1/\left(\sum_{i=0}^{t}\left(\begin{array}{c} n\\ i \end{array}\right)\times 2^{k}\right)$. Notice that the adversary is able to verify whether the guess is correct or not by rapidly substituting the keys into the verifier messages *V*_*R*_, sending it to *T*_*i*_, and validating the response *V*_*i*_. ISO standard 14443 specifies the data exchange rate between the reader and the tag, which is 106 kbit to 848 kbit [28]. Based on this data, we can calculate the relationship between the different codes, the amount of messages the tag transmitted, and the response time, where the response time is the time required for a tag to respond to reader's query. The result is depicted in Table 1.

Assume the adversary tries to launch the guessing attack by rapidly querying the tag before the tag's stored key can be updated by the valid reader. Generally, in real-world applications, the adversary is unable to rapidly query a specific tag for a long time because of the mobility of the tag's carrier. Therefore, attacks that require more than one hour may be regarded as useless. Nonetheless, the adversary may steal a tag from the system to avoid side effects caused by carriers. Nevertheless, in some existing RFID systems, tags will be recycled regularly. For example, in the public transportation systems, the RFID tagged tickets will be recycled and calculated every day. The system manager can find that if a tag has been stolen and remove that tag from the system. As a result, the stolen tag will be unusable hereafter, and the attacker can no longer threaten the system with the tag. In other words, if the required time of an attack is higher than one day, the system can be considered secure. In Table 2, we estimate the success probability of key guessing attack if the attacker performs the attack by rapidly querying the tag either within one hour or within one day. Based on the above arguments and analysis, we choose *C*(47,24,11), *C*(63,57,3), *C*(63,39,9), *C*(63,24,15), and *C*(127,36,39) as the candidates for implementing our protocol since they provide better security. In some systems with intensive surveillance, *C*(31,26,3) can also be taken into consideration.

### 5.6. Comparison

In the following, we show the comparisons between our protocol and other related protocols in terms of the security requirements. We take Chien's SASI protocol [29] and Chien-Laih's ECC-based protocol [30], Juels-Weis' HB^+^ protocol [19], and Sun-Ting's *Gen*2^+^ protocol [26] into comparison. These lightweight protocols are similar to our protocol in basic assumptions. The comparison results of security requirements are shown in Table 3.

SASI protocol was proposed in 2007. This ultralightweight authentication protocol requires only PRNG and simple bitwise operations which are supported by EPC Gen2 tags. However, studies [31, 32] showed that SASI is vulnerable to desynchronizing and tracing attacks. Chien-Laih's ECC-based lightweight authentication protocol was proposed in 2009. However, this protocol cannot defend against the tracing attacks [33]. Juels-Weis's HB^+^ protocol is a multiround lightweight mutual authentication protocol. It requires the tags and the readers to share the same secret to perform its authentication protocol. Studies have proved that HB^+^ protocol is vulnerable to a man-in-the-middle attack [21]. In this attack, the attacker can retrieve the entire secret and impersonate the valid tag. Therefore, HB^+^ cannot satisfy authenticity. And, without a secret key update scheme, this protocol also cannot maintain forward secrecy. Sun-Ting's Gen2^+^ protocol is another lightweight mutual authentication protocol suitable for Gen2 standard. In [27], the authors proved that the attacker can calculate a fake message to pass the authentication process by replaying the previous messages. As a result, Gen2^+^ is unable to fulfill authenticity requirement.

### 5.7. Summary

We had analyzed the security of our protocol and showed that our protocol provides high security against the common security threats of the RFID systems. We also analyzed the adversary's success probability of recovering the secret key. With careful parameter selection, the attacker will need a long time to break the protocol. Therefore, in most application scenarios, our protocol provides a good solution for securing the RFID system.

## 6. Evaluation

In this section, we will first describe the hardware constraints on selecting parameters for our lightweight protocol. Then we will have a discussion on the computational loads of the reader and the tag. Finally, based on the analysis, we will compare our protocol with previous works in terms of performance.

### 6.1. Parameter Selection

We analyze the memory storage and computational capability on the low-cost RFID tags in this section. Based on the analysis, we will select parameters that provide enough security to our protocol and show that the protocol is lightweight enough to be implemented on the tags.

Since our protocol requires tag to store the generator matrix *G* and the parity check matrix *H*, the size of the matrices should not exceed the size of the tag's storage memory. Fortunately, most passive RFID tags have 1 Kbytes–8 Kbytes of storage; some may even have up to 64 Kbytes of storage [15]. This is sufficient for storing our matrices, which only require about 1 Kbytes-2 Kbytes. With the secret keys and other information added, the requirement is still within the tag's capability.

Next we turn our attention to the tag's computational power. As estimated in [15], the cost of an RFID tag should range from US$0.05 to US$0.10, and the area of a silicon chip is limited to approximately 0.25 mm^2^–0.5 mm^2^. Under these constraints, the number of logical gates that can be mounted on the chip is limited. Researchers from Auto-ID Labs have estimated that only 400–4000 gate equivalents (GE) can be used for the security related functionality [3].

When running our protocol, the tag has to perform vector-matrix multiplication for decoding and encoding. According to [34], this multiplication can actually be performed by broadcasting columns of the matrix and multiplying them with the corresponding row elements of the vector. Therefore, the operation is simply to rapidly read a column of the matrix from the memory, XOR it with the vector, and accumulate them into a buffer until all the columns are multiplied. The only operation required in the vector-matrix multiplication is a bitwise XOR, which is not an obstacle for the RFID tags. However, during the operation, the elements need to be loaded into the registers. This implies that our protocol requires at least 3*n* bits of registers for buffer implementation. We also need *n* bitwise XOR logical gates for the multiplication. The other operations, like adding error vector, can also be performed by using these buffers and XOR gates. One bit register takes 6 GE to implement, and a XOR logical gate costs 2.67 GE. Besides, in our protocol, a one-way hash function is required to compute the verifier messages. Implementation of a lightweight hash function costs about 2500 GE [16]. Based on the above analysis, we now estimate the number of required GE for each parameter set we suggested in Section 5.5. The result is listed in Table 4. Most of these do not exceed the limitation of 4000 GE.

### 6.2. Performance

It is difficult to implement our protocol on the current low-cost RFID tags, since most of the RFID modules are not user-programmable. They run merely the processes that set in manufacturer phase. Therefore, we cannot evaluate the time consuming on the real tags. Hence, we calculated the average amount of transmitted messages in our protocol to estimate the average time of communicating.

Assume that a reader is going to authenticate a tag from *N* tags. We denote *L* as the length of the secret key. In our protocol, the secret key length *L* is equal to the length of the message, *n*. For each tag, it will send *k* ⊕ *C*_*i*_′, *V*_*i*_, and *N*_*i*_ to respond to the reader's single query. All of them are *L* bits in length. For the reader, it will broadcast *C*_*R*_′ and *N*_*R*_ to tags (2*L* bits). After receiving one response message from a tag, the reader will try to decode it. Whatever the decoding result is, the reader always sends a *L*-bit message *V*_*R*_ to the tag. Since the reader will receive at most *N*  responses from the tags, it will broadcast at most *NL* bits of *V*_*R*_ messages. As a result, the total amount of transmitted messages of the reader and the tags during the authentication process is *NL* + 2*L* and 3*NL*, respectively.

Now we can estimate the running time of our protocol. First note that all tags compute and transmit their messages in parallel; therefore, we should use the amount of total message of a single tag (3*L* bits) for our calculation. Also, based on the fact that the data rate specified in ISO 14443 standard is 106 Kbits to 848 Kbits, we can compute the required data transmitting time of our protocol. The result is shown in Table 5. Even in the worst case scenario, the longest transmitting time is still about 0.13 seconds, which is negligible for most users.

In order to minimize its computational load, the reader will attempt to filter out the unnecessary verifier messages *V*_*i*_. At step 3, when the tag discovers the syndrome *s* it computed is not matching with the syndrome pattern its stored, the tag will assign a random value to the masked codeword. Even though the probability is small, this random value may be recognized as a valid codeword by the reader. If a random number is recognized as the codeword, the reader has to verify an extra verifier message, thus adding its load. The probability can be computed by dividing the number of all possible *C*_*i*_′ by the number of all possible random values; that is, $\left(\sum_{i=0}^{t}\left(\begin{array}{c} n\\ i \end{array}\right)\times 2^{k}\right)/2^{n}$. In Table 6, we show the probability that the random number is recognized as a valid codeword between different codes. Note that *C*(31,26,3) and *C*(63,57,3) are perfect codes. Therefore, their probability of mistake is 1 since every random message can be corrected to a valid codeword.

Because the number of possible syndrome patterns is limited, a pattern might be shared by many tags. In other words, tags might store the same syndrome pattern. If the reader wants to authenticate one of these tags, each of them will respond with a valid codeword and verifier message. If that is the case, the reader will have to verify unnecessary verifier messages. The number of tags that share the same syndrome pattern is *N*/2^*k*^, if the syndrome patterns are randomly distributed to the tags. Taking the mistaking probability shown in Table 6 and the number of unnecessary responses into consideration, we estimate the average number of verifier messages *V*_*i*_ in which a reader has to verify in an authentication process. The result is shown in Table 7. The greater the number, the heavier the reader's computational load. Notice that the target tag might not be the one of these *N* tags in real-world applications; therefore, we have to remove the target tag from the experiment in order to get a fair result. Depending on the above evaluation, *C*(63,24,15), *C*(63,39,9), and *C*(127,36,39) provide good balance for the reader in both security and performance.

### 6.3. Comparisons

We compare the amount of transmitted messages between different authentication protocols as follows. Still taking Chien's SASI protocol [29] and Chien-Laih's ECC-based protocol [30], Juels-Weis's HB^+^ protocol [19], and Sun-Ting's *Gen*2^+^ protocol [26] into comparison, assume that the reader needs to authenticate a specific tag from a group of *N* tags. The amounts of messages sent between different number of tags and protocols are presented in Table 8.

In SASI protocol, the tags first send pseudonyms *IDS*s to the reader, and the reader replies with the messages *A*, *B*, and *C* to the target tag. Finally, the tag responds with message *D* to the reader. Each message is Kbits in length. In this protocol, the reader is able to find its target tag from the tags' responding *IDS*s. Therefore, the reader does not need to transmit any unnecessary message to the nontarget tags. In Chien-Laih's ECC-based protocol, the exchanged messages including a random number *N*_*R*_, the message sets $\left\{({\tilde{C}}_{i},{\tilde{V}}_{T}),({\hat{C}}_{i},{\hat{V}}_{T})\right\}$ and *V*_*S*_. ${\tilde{C}}_{i}$ and ${\hat{C}}_{i}$ are Kbits in length. On the other hand, *N*_*R*_, ${\tilde{V}}_{T}$, ${\hat{V}}_{T}$, and *V*_*S*_ are generated by a PRNG. We denote their length in bits as *D*. When the reader wants to find a tag from a group of tags, it has to authenticate every tag until it finds its target tag. In HB^+^ protocol, the reader and the tag exchange two random numbers and one bit message *z* in a single round. But the reader is still required to authenticate each tag to find its target tag. In Gen2^+^ protocol, the tag transmits 16-bit message set (*a*, *b*, *check*) to the reader, and the reader responds with 16-bit *ck*′ to the tag in a single round. After running *Q* rounds, the tag eventually responds with a 96- bit EPC to the reader. In this protocol, the reader has to authenticate each tag until it finds its target tag.

Compared with these protocols, the total amount of messages our protocol sent is no greater than most of the existing protocols. Although SASI protocol provides a very efficient identification mechanism based on tags' pseudonyms, the fixed pseudonyms make the tags vulnerable to tracing attack before they can be updated again.

## 7. Conclusion

Security and privacy issues on RFID have been studied in recent years due to the rapid growth of RFID systems. Many researchers worry about the disadvantages of RFID technology, such as keeping their location privacy and confidentiality of private information. On the other hand, manufacturers do not provide security functionality on their products because of the native limitation of RFID tags. As a result, researchers have proposed substantial lightweight authentication protocols for securing low-cost RFID tags.

Some real-world RFID application scenarios require a reader to find out and authenticate a tag from a group of tags. In previous works, the reader has to authenticate each tag individually until the reader found the target one, thus greatly increasing the communication and computation time. To address this problem, our protocol provides an error correction codes based mechanism to minimize the computational load of reader. When receiving query, the tags respond with verifier messages along with different codewords in which some of them cannot be decoded. The reader can filter out the unnecessary verifier messages by examining these codewords, therefore improving its performance.

In this paper, we presented a single-round lightweight mutual authentication protocol. The protocol is designed with decoding and encoding operations on error correction codes, pseudorandom number generating, and a hash function. These operations are proved lightweight enough to be implemented on low-cost RFID tags or can be realized by using simple bitwise operations. Based on the secrecy of shared keys, the reader and the tag can establish a mutual authenticity relationship. Further analysis of the protocol showed that it also satisfies integrity, forward secrecy, anonymity, and untraceability. Compared with other lightweight protocols, the proposed protocol provides stronger resistance to tracing attacks, compromising attacks, and replay attacks.

## Figures and Tables

**Algorithm 1:**
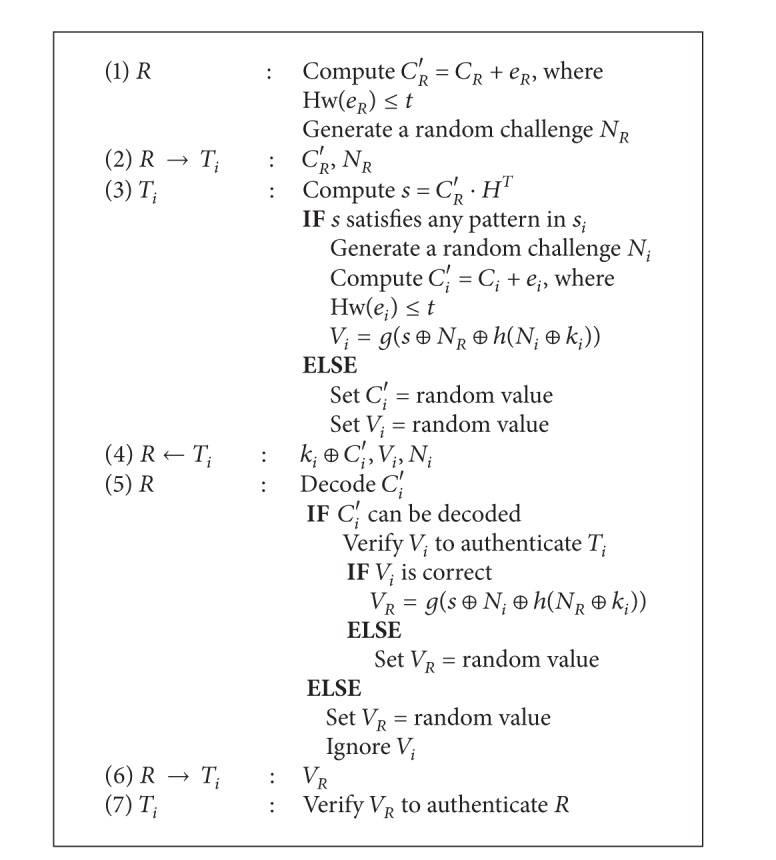
The proposed protocol.

**Algorithm 2:**
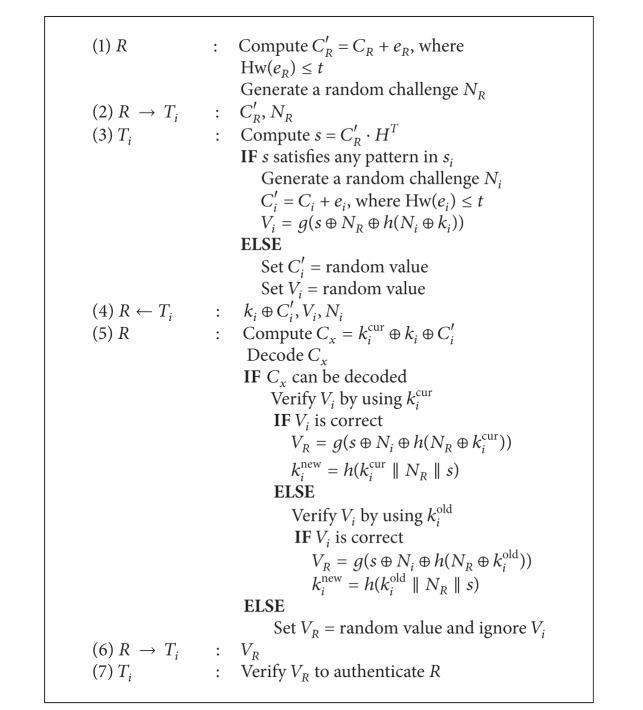
The proposed protocol with secret key updating.

**Table 1:** Estimated response time in different error correction codes.

Error correction code	Messages amount (bits)	Response time (μs)
*C*(7,4, 3)	21	24.8∼198.1
*C*(15,5, 7)	45	53.1∼424.5
*C*(24,12,8)	72	84.9∼679.2
*C*(31,26,3)	93	110.0∼877.4
*C*(31,6, 15)	93	110.0∼877.4
*C*(47,24,11)	141	166.3∼1330.2
*C*(63,57,3)	189	222.9∼1783.0
*C*(63,39,9)	189	222.9∼1783.0
*C*(63,24,15)	189	222.9∼1783.0
*C*(127,36,29)	381	449.3∼3594.3
*C*(255,187,19)	765	902.1∼7217.0

**Table 2:** Estimated success probability for key guessing attack.

Error correction code	Success probability of different attack periods	
	Within one hour	Within one day
*C*(7,4, 3)	1	1
*C*(15,5, 7)	1	1
*C*(24,12,8)	0.56∼1	1
*C*(31,26,3)	0.002∼0.02	0.05∼0.37
*C*(31,6, 15)	0.02∼0.14	0.43∼1
*C*(47,24,11)	9.3 × 10^−8^∼7.5 × 10^−7^	2.2 × 10^−6^∼1.8 × 10^−5^
*C*(63,57,3)	2.2 × 10^−13^∼1.8 × 10^−12^	5.3 × 10^−12^∼4.2 × 10^−11^
*C*(63,39,9)	5.8 × 10^−12^∼4.6 × 10^−11^	1.4 × 10^−10^∼1.1 × 10^−9^
*C*(63,24,15)	1.9 × 10^−10^∼1.5 × 10^−9^	4.6 × 10^−9^∼3.7 × 10^−8^
*C*(127,36,29)	8.3 × 10^−24^∼6.6 × 10^−23^	2.0 × 10^−22^∼1.6 × 10^−21^

**Table 3:** Comparison of security properties.

	Our Protocol	Chien's	Chien and Laih's	Juels and Weis's	Sun and Ting's
		[29]	[30]	[19]	[26]
Authenticity	*✓*	*✓*	*✓*	***✗***	***✗***
Integrity	*✓*	*✓*	*✓*	*✓*	*✓*
Forward secrecy	*✓*	*✓*	*✓*	***✗***	*✓*
Anonymity	*✓*	*✓*	*✓*	*✓*	*✓*
Untraceability	*✓*	***✗***	***✗***	*✓*	*✓*
Resistance to compromising	*✓*	*✓*	*✓*	*✓*	*✓*
Resistance to desynchronizing	*✓*	***✗***	*✓*	*✓*	*✓*

*✓*: satisfied; ***✗***: unsatisfied.

**Table 4:** Estimated gate equivalents for different parameters.

Error correction code	Required gate equivalents
*C*(31,26,3)	3141
*C*(47,24,11)	3471
*C*(63,24,15)	3802
*C*(63,39,9)	3802
*C*(63,57,3)	3802
*C*(127,36,39)	5125

**Table 5:** Estimated transmitting time for different parameters.

Error correction code	Required transmitting time (ms)		
	*N* = 1	*N* = 10	*N* = 100
*C*(31,26,3)	0.2∼1.8	0.5∼4.4	3.8∼30.7
*C*(47,24,11)	0.3∼2.7	0.8∼6.7	5.8∼46.6
*C*(63,24,15)	0.4∼3.6	1.1∼8.9	7.8∼62.4
*C*(63,39,9)	0.4∼3.6	1.1∼8.9	7.8∼62.4
*C*(63,57,3)	0.4∼3.6	1.1∼8.9	7.8∼62.4
*C*(127,36,39)	0.9∼7.2	2.2∼18.0	15.7∼125.8

**Table 6:** Probability of mistaking the random number as valid codeword.

Error correction code	Probability
*C*(31,26,3)	1
*C*(47,24,11)	0.206
*C*(63,24,15)	0.001
*C*(63,39,9)	0.038
*C*(63,57,3)	1
*C*(127,36,39)	9.1 × 10^−6^

**Table 7:** Estimated number of unnecessary verifier messages.

Error correction code	Average number of extra verifier messages		
	*N* = 1	*N* = 10	*N* = 100
*C*(31,26,3)	1	10	100
*C*(47,24,11)	0.2	2.1	20.6
*C*(63,24,15)	1.1 × 10^−3^	1.1 × 10^−2^	0.1
*C*(63,39,9)	3.8 × 10^−2^	0.4	3.8
*C*(63,57,3)	1	10	100
*C*(127,36,39)	9.1 × 10^−6^	9.1 × 10^−5^	9.1 × 10^−4^

*N*: number of tags.

**Table 8:** Comparison of total messages transmitted.

Authentication protocol	Total amount of transmitted messages (bit)		
	*N* = 1	*N* = 10	*N* = 100
Our protocol	6*L*	42*L*	402*L*
Chien's SASI [29]	5*L*	14*L*	104*L*
Chien and Laih's ECC-based [30]	4*D* + 2*L*	40*D* + 20*L*	400*D* + 200*L*
Juels and Weis's HB^+^ [19]	*Q* × (1 + 2*L*)	10*Q* × (1 + 2*L*)	100*Q* × (1 + 2*L*)
Sun and Ting's Gen2^+^ [26]	32*Q* + 96	10 × (32*Q* + 96)	100 × (32*Q* + 96)

*N*: number of tags; *L*: key length; *Q*: number of rounds; *D*: length of random number.

## Notation

*S*: RFID backend server
*R*: RFID reader
*T*_*i*_: A RFID tag
*s*: Syndrome pattern
*s*_*i*_: A syndrome pattern of *T*_*i*_
*k*_*i*_: A secret key of *T*_*i*_
*g*(): Pseudorandom number generator
*h*(): One-way hash function
*G*: Generator matrix
*H*: Parity check matrix
*C*_*R*_, *C*_*i*_: ECC codeword from *R* and *T*_*i*_, respectively
*e*_*R*_, *e*_*i*_: ECC error vector from *R* and *T*_*i*_, respectively
*V*_*R*_, *V*_*i*_: Verifier message from *R* and *T*_*i*_, respectively
*N*_*R*_, *N*_*i*_: Random nonce from *R* and *T*_*i*_, respectively
Hw(): Hamming weight.
